# Familial Hypercholesterolaemia as a Predisposing Factor for Atherosclerosis

**DOI:** 10.3390/biomedicines10102639

**Published:** 2022-10-20

**Authors:** Anastasia V. Poznyak, Larisa Litvinova, Paolo Poggio, Alexander N. Orekhov, Alexandra A. Melnichenko

**Affiliations:** 1 Institute for Atherosclerosis Research, Osennyaya 4-1-207, Moscow 121609, Russia; 2 Center for Immunology and Cellular Biotechnology, Immanuel Kant Baltic Federal University, 6 Gaidara Street, Kaliningrad 236001, Russia; 3 Unit for Study of Aortic, Valvular and Coronary Pathologies, Centro Cardiologico Monzino IRCCS, Via Carlo Parea 4, 20138 Milan, Italy; 4 Laboratory of Angiopathology, Institute of General Pathology and Pathophysiology, 8 Baltiiskaya Street, Moscow 125315, Russia

**Keywords:** lipid, cholesterol, hypercholesterolaemia, atherosclerosis

## Abstract

Lipid metabolism alterations are an important component of the pathogenesis of atherosclerosis. However, it is now clear that the atherogenesis process involves more than one mechanism, and more than one condition can predispose this condition. Multiple risk factors contribute to the atherosclerosis initiation and define its course. Familial hypercholesterolaemia is a disorder of lipid metabolism that often leads to atherosclerosis development. As is clear from the disease name, the hallmark is the increased levels of low-density lipoprotein cholesterol (LDL-C) in blood. This creates favourable conditions for atherogenesis. In this review, we briefly described the familial hypercholesterolaemia and summarized data on the relationship between familial hypercholesterolaemia and atherosclerosis.

## 1. Introduction

Being an autosomal co-dominant disorder, familial hypercholesterolaemia (FH) is characterized by increased levels of low-density lipoprotein cholesterol (LDL-C) in the blood. This condition leads to a 3–13 times higher chance of premature atherosclerosis development compared to people with normal LDL cholesterol levels [1]. Two forms of the disorder have been recognized. The heterozygous form is characterized by the presence of one mutation in low-density lipoprotein receptor [LDLR] and apolipoprotein B [APOB] genes and gain-of-function mutations in the proprotein convertase subtilisin/kexin type 9 [PCSK9] gene), and the presence of two mutated alleles is responsible for a more severe homozygous FH (HoFH) phenotype. The heterozygous form is diagnosed based on two to three times higher LDL-C concentrations in blood compared to normal, while the corresponding rate for homozygous hypercholesterolaemia can be up to ten times higher. Although, in some regions, founder effects can affect the statistics; on average, between 0.17% to 0.5% of people suffer from a heterozygous form of the disease. Research shows that homozygous hypercholesterolaemia, which was said to affect 0.001% of the population, turns out to be around three times more common [2].

The risk for atherosclerotic cardiovascular disease (ACD) is greatest among patients with the homozygous form of the disorder. However, recent research based on molecular diagnosis shows that, in some cases, the LDL-C concentrations for some of the individuals with the heterozygous mutation are higher than for those with the homozygous form [3]. This means that the risk of ACD for these patients is also very high; and vice versa: some individuals with a molecularly diagnosed homozygous form of the disorder demonstrate lower LDL-C levels (≥5–10 mmol/L [≥190–400 mg/dL]), initially associated with the heterozygous form [4].

The phenotypic heterogeneity can be explained by the fact that the LDL-C concentrations depend on both the rare, large-effect monogenic variants and common, small-effect gene variants, which makes the classification even more complex [5]. Since the main predisposing factor for the development of early atherosclerotic hypercholesterolaemia is the LDL-C concentrations rather than the molecularly diagnosed genotypic form of the disorder, the best medical practice requires defining the severe phenotype of the disorder to identify individuals at the highest risk for atherosclerotic hypercholesterolaemia across patient groups with both homozygous and heterozygous forms [6].

The risk for ACD is greatest among the following groups: patients with familial hypercholesterolaemia and previous symptoms of ACD, individuals suffering from advanced subclinical atherosclerosis, and patients with LDL-C levels over 8 mmol/L (310 mg/dL) [7]. Identifying such cases is important for choosing a more cost-effective treatment. In some cases, standard easily available lipid-lowering statins and ezetimibe can be effective. If the patient has persistent raised LDL-C concentrations despite the treatment and thus is deemed at higher risk for ACD, more efficacious but more expensive treatment with newer drugs can be considered, for example, mipomersen, lomitapide, and PCSK9 inhibitors, of which the latter turns out to be the most cost-efficient [8].

We already know that higher ACD risk arises from chronic exposure to raised LDL-C in the blood. Since LDL-C concentrations, which directly result in a higher risk for ACD, vary across the previously classified forms—non-mutated, heterozygous, or homozygous—this categorization does not reflect the actual risk of atherosclerosis for the patients. Considering the availability of modern and more efficient cholesterol-lowering drugs and insufficient evidence based on the genetic heterogeneity of the disorder specifically, the International Atherosclerosis Society convened an expert panel to form consensual treatment recommendations for patients at the highest risk for ACD [9].

## 2. The Pathophysiology of Familial Hypercholesterolemia

FH is an autosomal co-dominant inherited condition caused by an alteration in genes encoding the LDL receptor (LDLR), apolipoprotein B (APOB), and proprotein convertase subtilisin/kexin type 9 (PCSK9). The most common is the mutation in the LDLR gene on chromosome 19 [10]. Over a thousand mutation types have been identified so far. Clinical expression and the LDLRs activity levels allow for a two-type classification of the cases: the “receptor-negative” (<2% LDLR activity compared to normal) and the “receptor-defective” (2–25% LDLR activity). There is an inverse correlation between LDLR activity and LDL-C concentrations, which means that lower activity of LDLRs results in reduced LDL-C uptake [11]. This leads to three to six times higher LDL-C concentrations in patients with homozygous FH compared to normal, with total cholesterol levels at >500 mg/dL. Individuals with heterozygotes inherited from only one of the parents, however, demonstrate only two to three times higher LDL-C levels compared to normal. The LDL-C concentrations may vary depending on the type of mutation and environmental factors (total cholesterol at >300 mg/dL) [12].

## 3. Familial Hypercholesterolemia and Cardiovascular Disease

**Figure 1:**
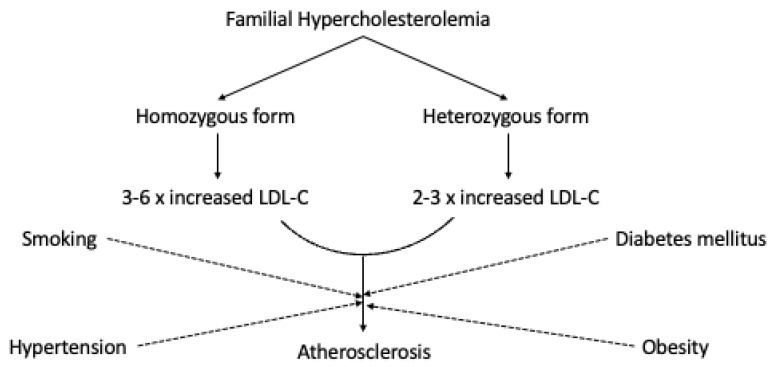
The potential relationship between FH and atherosclerosis.

Individuals with homozygous familial hypercholesterolemia demonstrate cardiovascular manifestation before the age of 10. The symptoms are very specific, including sudden death. Individuals suffering from receptor-negative FH may die prematurely before the age of 20 without proper treatment, while patients with the receptor-defective form develop clinically significant CVD before they reach 30 [13]. The cholesterol concentrations and the subjects’ clinical pattern depends on the patient’s genetics. The level of abnormality in LDLR genes also affects the natural course considerably for heterozygous FH patients, as well as various risk factors such as smoking, hypertension, and diabetes mellitus [14]. The risk of myocardial infarction before the age of sixty in unidentified FH patients is 60% higher in men and 30% higher in women [15]. In Figure 1, we schematically represent the potential relationship between FH and atherosclerosis.

## 4. Genetic Variables in CV Risk in FH Population

There is much evidence proving the existence of an impact of various genetic parameters on the disease phenotype and CVD risk in the FH population. This applies to genetic variants and SNPs in various genes, including those controlling LDL metabolism, oxidative stress, lipoprotein metabolism, renin angiotensin aldosterone system, and other processes [16]. Khera et al. have found that carriers of an identified FH-causing mutation who suffer from severe hypercholesterolemia had an enhanced CVD risk in comparison to severe hypercholesterolemia patients who did not carry the identified mutation. This suggests that mutation status beyond LDL-C may influence CVD prognosis in the FH population [17]. The influence of various FH-causing mutations on the disease severity and CVD risk is not clear. Among carriers of LDLR mutations, the ones carrying LDLR-negative mutations were found to be more vulnerable to premature atherosclerosis development than patients with LDLR-defective mutations [2].

Telomere length in somatic cells is also an emerging genetic parameter that can help predict more vulnerability for CVD and severe disease phenotype FH patients. However, whether telomere length, as a biological index of accelerated cellular aging, may also correlate with disease severity phenotype, risk of premature onset of CVD, life expectancy, or mortality in HeFH patients has not been investigated [18]. Future studies should address this issue to clarify whether this genetic parameter may be useful for CVD risk stratification in the HeFH population.

## 5. LDL Cholesterol

Prospective observational studies, whole genome association studies, and Mendelian randomization studies have shown that hypercholesterolaemia is an independent predisposing factor for ACD [19,20]. Several clinical experiments, including surrogate interventional trials of lipid-lowering drugs—mainly statins—have reported a reduction in clinically significant cardiovascular symptoms and mortality, which proves that hypercholesterolaemia can be a cause of ACD [21].

Being exposed to extreme LDL cholesterol concentrations in blood, homozygous FH patients are at 100 times higher risk for premature ACD compared to individuals with normal LDL-C levels. These patients often demonstrate aortic or supra-aortic valve stenosis as well as aortic atherosclerosis and atherosclerosis in coronary, carotid, and peripheral arteries [22].

The pathogenic correlation between long-term exposure to increased LDL-cholesterol concentrations and premature atherosclerosis in individuals with homozygous FH can be illustrated by the “cholesterol-year score”—a marker that evaluates the exposure to high cholesterol over time [23]. Increased LDL-C concentrations result in worse clinical courses, which applies to patients with both homo- and heterozygous forms of the disorder. Untreated FH affects younger people in particular. Before the discovery of statins, adjusted mortality rates for FH patients were 125 times higher for females and 48 times higher for males between 20 and 29 years, compared with individuals with normal LDL-C levels [24]. In recent research, Do et al. performed exome sequencing in the protein-coding regions of 9793 genomes from individuals with early myocardial infarction and reported that LDLR mutations were the cause in 2% of the cases [25]. In a similar study, Nanchen and colleagues also found out that 5% (70 of 1451) of patients suffering from acute coronary syndrome before the age of 60 had clinically diagnosed or probable FH [26].

Increased risk of premature mortality and early ACD is disapprovingly reported even for statin-treated patients with homozygous FH [27]. Raal and co-workers evaluated the occurrence of major ACV events in 149 homozygous FH patients from South Africa. After the introduction of statins in South Africa in 1990, the number of such events decreased by 51% [28]. However, the age by which the subjects had had the first major vascular event was only delayed from 12.8 years to 28.3 years on average. Nine of ten patients have suffered at least one major vascular event by the age of 40 [29]. A report by Thompson and colleagues shares the long-term outcomes of homozygous FH patients treated at the Hammersmith Hospital in London, UK, over 50 years [30]. Individuals who died or survived during the follow-up period were compared. The surviving patients were more frequently treated with statins and apheresis. The care quality improved significantly throughout this period. Even so, the occurrence of ACD cases was still high in survivors: 33% demonstrated aortic stenosis, 14% required aortic valve replacement, and 37% suffered from coronary heart disease. Both studies report very high total cholesterol levels even during the treatment (13.1 mmol/L (505 mg/dL) in the patients from South Africa and 8.1 mmol/L (320 mg/dL) in the UK cohort). The results of these studies prove that increased cholesterol level has a causal role in ACD development in homozygous FH patients and the urgent need for treatment in these individuals.

Greater ACD risk in many heterozygous FH patients can be explained by high LDL-C concentrations that are refractory to cholesterol-lowering drugs. Pre-treatment LDL-C levels above 8 mmol/L (310 mg/dL) can be a sign of a more severe heterozygous phenotype independent of other traditionally recognized risk factors (diabetes mellitus, smoking, hypertension, or a family history of premature ACD) [31]. However, these factors contribute to increased total risk. In a Dutch study of FH patients, one in three thousand people, or 0.11 of the Dutch cohort with FH, demonstrated high LDL cholesterol concentrations. These were associated with an odds ratio of 1.36 (95% CI 1.09–1.69) in the pre-treatment period, compared with individuals with lower levels of LDL cholesterol [32]. LDL cholesterol levels above 8 mmol/L (310 mg/dL) that are refractory to maximally tolerated statin treatment in patients without earlier symptoms of atherosclerotic hypercholesterolaemia are suggested to be an indication for reimbursement of apheresis (either selective lipoprotein apheresis or plasmapheresis) [33].

When evaluating the severity of the FH phenotype, the subject’s age at treatment initiation should be considered. Since the arterial walls have been long exposed to high LDL cholesterol concentrations in patients who have received late treatment (for example, after the age of 40), a greater risk of ACD occurs in such cases [34].

Most guidelines recommend that LDL cholesterol should be reduced by 50% in FH patients. Sometimes more specific absolute levels are mentioned, for example, concentrations less than 2.5 mmol/L (100 mg/dL), or 1.8 mmol/L (70 mg/dL) in subjects with ACD [35].

A cross-sectional study involving 1249 Dutch patients with heterozygous FH reported that only 261 (21%) of 904 (70%) subjects who received lipid-lowering treatment aimed at reducing LDL-C levels by 50% reached LDL cholesterol levels below 2.5 mmol/L (100 mg/dL) [36]. The SAFEHEART study involved 2170 patients with molecularly defined heterozygous FH treated in Spain over 5 years on average—1562 (72%) subjects received maximum lipid-lowering statin treatment aimed to reduce LDL-C by at least 50%—alone or in combination with ezetimibe [37]. Of these, only 247 (11%) participants attained the target LDL-C level below 2.5 mmol/L (100 mg/dL). Among 277 patients with earlier diagnosed ACD, only 13 (5%) reached an LDL-C level less than 1.8 mmol/L (70 mg/dL). These findings confirm the huge deficit in controlling lipid concentrations in FH patients, especially for the secondary prevention of ACD.

## 6. Currently Used Treatment Strategies for Familial Hypercholesterolemia

A number of major clinical studies of FH patients have been carried out.

### 6.1. Non-Pharmacological Treatment

Lifestyle correction is beneficial for all familial hypercholesterolaemia patients. Thus, regular physical activity, smoking cessation, and eating habits correction can improve the course of the disease.

Considering the paediatric population diet, correction should be started after two years of age. A healthy diet rich in whole grains, fish, vegetables, and fruits is suggested. Data on other dietary supplements, including omega-3 or dietary fibres, are scarce and inconclusive.

### 6.2. Lipoprotein Apheresis

Lipoprotein apheresis is a selective approach to the traditionally applied plasmapheresis, which can be used as an addition to pharmacotherapy. However, it is expensive and time-consuming. The single treatment was shown to lower the level of LDL-C by 55–70%, and the long—term therapy can lead to xanthomas and plaque regression, which improves the cardiovascular events prognosis. However, there are side effects, such as iron deficiency, hypocalcaemia, abdominal pain, and hypotension. Due to the hypotension risk, the use of lipoprotein apheresis should not be combined with any antihypertensive drugs. The lipoprotein apheresis can be started from the age of 5 and no later than the age of 8 years.

### 6.3. Pharmacotherapy

The ASAP, “Atorvastatin vs. Simvastatin on Atherosclerosis Progression”—an interventional statin treatment trial—involved 325 FH patients [38]. The high-dose regimen of atorvastatin 80 mg was compared with the conventional dose regimen of simvastatin 40 mg.

Among the simvastatin-treated subjects, increased carotid intima-media thickness (CIMT) was reported, while the atorvastatin-treated subjects presented decreased CIMT. The LDL-C levels were reduced by 50% in the atorvastatin-administered patients and by 40% in the simvastatin-administered group.

The Ezetimibe and Simvastatin in Hypercholesterolemia enhance atherosclerosis regression (ENHANCE) study carried out in 2008 assessed the effect of a single simvastatin 80 mg regimen compared to combined treatment with both simvastatin 80 mg and ezetimibe 10 mg [39]. The trial involved 720 patients with FH. The simvastatin-administered group showed a 41% reduction in LDL-C levels, while the corresponding result for the group receiving combined treatment was 58%. The patients with a combination therapy also revealed a 26% decrease in high sensitivity-C-reactive protein (CRP). Despite the anticipation, no difference in CIMT was noticed between the two groups. This led to doubts about whether the CIMT is reasonable and the anti-atherosclerotic effect in interventional clinical trials for FH patients.

Several factors should be considered when analysing and comparing the results of the failed ENHANCE study and the successful ASAP study. We summarized the main findings of these two trials in Table 1. In the first study, nearly 90% of the patients had already been receiving statin treatment by the time they participated in the study, and their average CIMT was unexpectedly 0.69, which is a normal level. The CIMT might have already been stabilized by statin administration before the study. The average CIMT in ASAP study patients before the trial was 0.925, which explains a clearer reduction after drug intervention.

The additive effect of ezetimibe was not explained in the ENHANCE study. The Study of Heart and Renal Protection and the Improved Reduction of Outcomes: Vytorin Efficacy International Trial provides more information about the effect of ezetimibe. For now, the following treatment principle has been suggested: difficulties in achieving the target LDL-C levels in patients with FH, as well as the unavailability of high-dose statin treatment due to side effects, is an indication for concurrent ezetimibe administration.

## 7. Efficacy of Ezetimibe in the Treatment of FH

While dietary management and statin therapy are quite effective in treating general hypercholesterolemia, FH can be refractory to these methods. Concurrent ezetimibe treatment may be more effective in such cases due to the specific pharmacological action of ezetimibe that suppresses cholesterol intake in the intestine [40]. However, for subjects with homozygous FH, only a 30% decrease in LDL-C concentrations can be achieved even with combined therapy, so LDL apheresis or liver transplantation can also be considered in such cases [41].

Dietary management can lead to cholesterol reduction by 10–15% in subjects with heterozygous FH, and the target level can sometimes be achieved through high-dose statin treatment. Since a statin-dose reduction may be needed due to the side effects such as myalgia, efficient combined treatment with less adverse reaction should be considered. In such cases, ezetimibe demonstrates an excellent additive effect, reducing the LDL-C concentrations by an additional 10–20% in patients with FH [42].

## 8. Statins

Statins are the first-line therapy in FH treatment. Currently, the guidelines suggest maximally tolerated statin doses for adults, though statin-only treatment rarely allows one to attain the target LDL-C concentrations. Considering the pharmacological mechanism of statins, which partly decrease the LDL-C levels by stimulating LDLR expression in the liver, it is expected that patients suffering from homozygous FH with null LDLR gene mutations would be refractive to such therapy [43]. However, these subjects respond to statin therapy but a lower degree than subjects with other FH types. This can be explained by the fact that the pharmacological mechanism of statins may vary, and sometimes they would act through VLVD (and thus LDL) synthesis suppression. The reported LDL-C lowering effect in such patients is lower than in non-FH subjects (~20% vs. 40–60%) [44].

Guidelines recommend initiating the statin treatment of children starting from the age of eight to ten years. The doses should be increased gradually until the target LDL-C level is reached. The safety of statin therapy in children is often questioned, though a meta-analysis of six studies involving 798 children treated with statins reported a considerable reduction in LDL-C levels (weighted mean difference: −30%), total cholesterol (−23%) and apoB (−25%) [45]. The report showed no major difference between the statin- and placebo-treated groups regarding side effects, muscle or liver toxicity, and sexual maturation. Rosuvastatin was not among the trialled statin agents in the meta-analysis [46]. However, its efficacy has also been assessed in HeFH children and adolescents. The study revealed a 35–45% decrease in LDL-C concentration, a much lower CIMT, and no adverse reactions affecting physical development or sexual maturation after 2 years of therapy. The mean LDL-C reduction level in HoFH subjects was 22.3%. Patients with residual LDLR showed the greatest LDL-C decrease at 23.5%, and the response rate for subjects with two LDLR null mutations was a 14% decrease [47].

Pitavastatin has also decreased LDL-C concentrations in patients between 6 and 17 years. There was reported no significant difference between the statin—and placebo-treated groups regarding adverse effects. Furthermore, a retrospective study involving HeFH patients has revealed a 44% reduction in the risk of coronary artery disease and mortality in subjects treated with moderate-to-high-intensity statin therapy. However, there are still not enough prospective studies of statin’s effect on cardiovascular outcomes in patients with FH [48].

A recent study involving consecutive HeFH patients treated with maximal tolerant doses of lipid-lowering therapy (median treatment and follow-up duration—9.5 years) reported the following results: although the LDL-C concentrations decrease after statin monotherapy and sometimes even reach the target levels, FH patients have the greater residual risk of CVD after statin-only treatment [49]. The study revealed that LDL-C concentrations in these patients exceeded the levels endorsed in current guidelines. Moreover, the subjects exhibited higher Lp(a) levels that could not be controlled by statins and thus increased the residual risk. These results prove the need for a combined therapy approach with drugs reducing LDL-C concentrations through different pharmacological mechanisms [50].

## 9. PCSK9 Inhibitors

Proprotein convertase subtilisin/kexin type 9 (PCSK9) is an enzyme primarily expressed in the liver that regulates the LDLR activity by binding LDLR on liver cells and promoting LDLR degradation [51]. As a result, increased PCSK9 concentrations cause hypercholesterolaemia. Gain-of-function abnormalities in the PCSK9 gene result in FH and a greater risk of ACD, while loss-of-function abnormalities are a cause of lower LDL concentrations in blood and reduced risk of coronary heart disease. This means that hypercholesterolaemia treatment can be based on suppressing the PCSK9 [52,53]. There are different types of PCSK9 inhibitors. Monoclonal antibodies such as alirocumab and evolocumab bind to circulating PCSK9, preventing it from interacting with LDLR, while siRNA molecules such as inclisiran suppress PCSK9 synthesis in the cells. Many different clinical trials of monoclonal antibodies in patients with hypercholesterolaemia, including individuals with FH, have been carried out [54].

### 9.1. Inclisiran

Inclisiran is an artificial molecule of small interfering ribonucleic acid, the molecular stability of which is supported by a range of modifications. Being administered via subcutaneous injections, this drug specifically binds to the messenger RNA of the PCSK9 gene promoting its degradation, which, in turn, leads to the persistent reduction of the PCSK9 protein levels, both intracellular and extracellular [55]. A single dose of inclisiran was shown to significantly reduce the circulating level of PCSK9 and LDL-C level in a dose-dependent manner compared to a placebo. These results were shown among patients with FH during the randomized, placebo-controlled phase 1 trial. Mentioned effects lasted 180 days after the administration. No severe adverse effects were observed, and only light and moderate cases were admitted [56].

In phase 2, placebo-controlled, randomized study ORION-1, 501 patients with hypercholesterolaemia and high cardiovascular risk received a single dose of inclisiran, which caused a dose-dependent lowering of LDL-C levels by up to 42% at day 180. The administration of two doses of inclisiran, at day 1 and 90, led to LDL-C levels lowering at day 180 by up to 53%. Moreover, the levels of very-low-density lipoprotein cholesterol, non-high-density lipoprotein cholesterol (non-HDL-C), and apolipoprotein B (apoB) levels appeared to be persistently decreased as well [57]. It is worth noting that both patients with diagnosed diabetes mellitus and non-diabetic individuals had a similar lowering in LDL-C and other atherogenic lipoproteins. There were no differences in serious adverse effects rates between the test and placebo groups. After one year of follow-up, no changes in adverse effects rates, and the time-averaged lowering of LDL-C levels was up to 39% in those who received one dose and up to 46% in those who received two doses. It was also shown that inclisiran can be used without dose adjustment and is equally effective and safe in patients with renal impairment. What is more, inclisiran did not affect platelets and white blood cells number and the inflammatory marker’s interleukin-6 and tumor necrosis factor α [57].

Recently, the results of phase 3 trials of inclisiran were published. ORION-9 enrolled 482 patients with heFH, who received 300 mg of inclisiran at days 1, 90, and 270. 450 participants exhibited a 48% decrease in the level of LDL-C after 510 days from the administration compared to the placebo group. The genotype of heFH had no impact on the inclisiran effectiveness. Lipoprotein (a) levels appeared to be reduced by 17% more in the inclisiran group than the placebo one, while the serious adverse effects rates were similar in the two groups [58].

In the ORION-10 trial (1561 individuals with diagnosed cardiovascular diseases) and the ORION-11 trial (1617 individuals with diagnosed cardiovascular diseases), the reduction of LDL-C levels was observed at day 510 by 52% and 50%, respectively, in comparison with placebo after administration of inclisiran 300 mg at day 1, day 90 and every 6 months over 540 days. Lipoprotein (a) levels were also decreased by inclisiran by 26% and 19% more than placebo, respectively. Among patients treated with inclisiran, injection-site adverse reactions were more frequent, but these reactions were generally mild, and none were severe or persistent. The rate of other adverse events was similar in the inclisiran and placebo groups [59].

### 9.2. Bile Acid Sequestrants

Drugs of this group decrease plasma levels of LDL-C by removing the bile acids from the enterohepatic cycle via binding them in the intestine. This leads to the increased bile acid formation from intrahepatic cholesterol, which, in turn, reduces intrahepatic cholesterol concentration and stimulates the upregulation of LDLR on the surface of the hepatocyte. Bile acid sequestrants can reduce LDL-C levels by 10–20%, but they often cause abdominal pain, constipation, nausea, and other side effects. Also, they interfere with the absorption of folate and fat-soluble vitamins, and appropriate dietary supplementation is recommended [60].

Recently, second-generation bile acid sequestrant colesevelam was evaluated in children with FH. This compound can be used in lower doses because of its greater affinity to bile acids. This reduces the side effects rate and makes the drug more tolerable. Now, colesevelam is the only bile acid sequestrant that is approved for the treatment of paediatric patients with HeFH [61].

### 9.3. Probucol

Probucol is a well-known anti-hyperlipidemic drug that was developed to manage coronary artery disease via its antioxidative properties. In recent years, it appeared to be effective in various cardiovascular diseases in humans and showed a variety of pharmacological effects. Moreover, apart from a variety of therapeutic effects, diversity in the mechanisms of action of probucol was also demonstrated. It is known that probucol can stimulate cholesterol efflux and increase reverse cholesterol transport by activation of CETP and scavenger receptor class B type I. Through these mechanisms, HDL-C levels can be decreased. Additionally, probucol was shown to decrease the risks of coronary artery disease development among patients with heterozygous FH. This drug has a big, although underestimated, potential for FH management [62,63].

## 10. Combined Therapy

Familial hypercholesterolaemia is a complex condition that usually requires more than one therapeutic approach [64]. The on-time start of therapy is crucial for a favourable prognosis. The main goal of this therapy is to avoid the development of atherosclerotic cardiovascular disease. The first part of the combined therapy is therapeutic diet modification and exercise. Briefly, total fat, saturated fat, trans-fatty acid, cholesterol, carbohydrate, sugar, and alcohol intake needs to be limited, and their limits are suggested. At the same time, consuming fibre-rich food, whole- and multigrain food, vegetables, fish, and fresh fruits is recommended. Regarding exercise, the performance of aerobic exercise 4–6 times a week and resistance exercises at least 2 times a week is advised [65].

However, further correction of LDL-C level is often needed, so pharmacological treatment is in charge. The first-line therapy is statins. Drugs of this group are usually administered at a high intensity, but even this is often not enough. So, the second-line choice, according to guidelines, is ezetimibe. PCSK9 inhibitors can be added if patients do not achieve the target with a maximal tolerable dose of statin plus ezetimibe [44].

## 11. Conclusions

In this review, we distinguished the relationship between atherosclerosis and familial hypercholesterolaemia. As a disorder associated with impaired lipid metabolism, familial hypercholesterolaemia cannot but affect the risks of atherosclerosis, as well as associated cardiovascular diseases and events. After analysing the data available in the literature on the relationship between atherosclerosis and familial hypercholesterolaemia, we concluded that these two diseases are inextricably linked, and familial hypercholesterolaemia can serve as a prerequisite for the development of atherosclerosis. This is realized due to a violation of lipid metabolism, which is the central event of familial hypercholesterolaemia and one of the main mechanisms of atherogenesis. A review of potential treatment modalities that largely overlap for these two diseases deserves special attention. Lifestyle corrections, including physical activity, smoking cessation, and diet, beneficially affect the health of familial hypercholesterolaemia patients. Additionally, beneficial effects were admitted for the use of lipoprotein apheresis and, of course, pharmacological treatment. Among effective drugs, statins, PCSK9 inhibitors, and ezetimibe should be noted. These lipid-lowering drugs also have the potential for the treatment of atherosclerosis.

## Figures and Tables

**Table 1:** Simvastatin and atorvastatin comparison.

Compound	Trial	Effect	References
simvastatin 40 mg	ASAP	increased CIMT; 40% reduced LDL-C levels	[36]
atorvastatin 80 mg	ASAP	decreased CIMT; 50% reduced LDL-C levels	[36]
simvastatin 80 mg	ENHANCE	41% reduced LDL-C levels	[37]
simvastatin 80 mg and ezetimibe 10 mg	ENHANCE	58% reduced LDL-C levels; 26% decreased hsCRP	[37]

## Data Availability

Not applicable.

## References

[1] Viigimaa M., Heinsar S., Lovic D., Katsimardou A., Piperidou A., Duishvili D. (2018). New Horizons in the Pathogenesis, Pathophysiology and Treatment of Familial Hypercholesterolaemia. Curr. Pharm. Des..

[2] Moradi A., Maleki M., Ghaemmaghami Z., Khajali Z., Noohi F., Moghadam M.H., Kalyinia S., Mowla S.J., Seidah N.G., Malakootian M. (2021). Mutational Spectrum of *LDLR* and *PCSK9* Genes Identified in Iranian Patients With Premature Coronary Artery Disease and Familial Hypercholesterolemia. Front Genet..

[3] Vrablik M., Tichý L., Freiberger T., Blaha V., Satny M., Hubacek J.A. (2020). Genetics of Familial Hypercholesterolemia: New Insights. Front. Genet..

[4] Bouhairie V.E., Goldberg A.C. (2015). Familial Hypercholesterolemia. Cardiol. Clin..

[5] Weissglas-Volkov D., Pajukanta P. (2010). Genetic causes of high and low serum HDL-cholesterol. J. Lipid Res..

[6] Futema M., Taylor-Beadling A., Williams M., Humphries S.E. (2021). Genetic testing for familial hypercholesterolemia—past, present, and future. J. Lipid Res..

[7] Bjelakovic B., Stefanutti C., Reiner Z., Watts G.F., Moriarty P., Marais D., Widhalm K., Cohen H., Harada-Shiba M., Banach M. (2021). Risk Assessment and Clinical Management of Children and Adolescents with Heterozygous Familial Hypercholesterolaemia. A Position Paper of the Associations of Preventive Pediatrics of Serbia, Mighty Medic and International Lipid Expert Panel. J. Clin. Med..

[8] Korman M., Wisløff T. (2018). Modelling the cost-effectiveness of PCSK9 inhibitors vs. ezetimibe through LDL-C reductions in a Norwegian setting. Eur. Hear. J.-Cardiovasc. Pharmacother..

[9] Alonso R., De Isla L.P., Muñiz-Grijalvo O., Diaz-Diaz J.L., Mata P., Foundation M.S.F.H. (2018). Familial Hypercholesterolaemia Diagnosis and Management. Eur. Cardiol. Rev..

[10] Civeira F.F., De Castro-Orós I., Pocoví M. (2010). The genetic basis of familial hypercholesterolemia: Inheritance, linkage, and mutations. Appl. Clin. Genet..

[11] Varret M., Abifadel M., Rabès J.-P., Boileau C. (2008). Genetic heterogeneity of autosomal dominant hypercholesterolemia. Clin. Genet..

[12] McGowan M.P., Dehkordi S.H.H., Moriarty P.M., Duell P.B. (2019). Diagnosis and Treatment of Heterozygous Familial Hypercholesterolemia. J. Am. Hear. Assoc..

[13] Safarova M.S., Kullo I.J. (2016). My Approach to the Patient With Familial Hypercholesterolemia. Mayo Clin. Proc..

[14] González-Lleó A.M., Sánchez-Hernández R.M., Boronat M., Wägner A.M. (2022). Diabetes and Familial Hypercholesterolemia: Interplay between Lipid and Glucose Metabolism. Nutrients.

[15] Zhao P.J., Ban M.R., Iacocca M.A., McIntyre A.D., Wang J., Hegele R.A. (2019). Genetic Determinants of Myocardial Infarction Risk in Familial Hypercholesterolemia. CJC Open.

[16] Maștaleru A., Cojocariu S.A., Oancea A., Constantin M.M.L., Roca M., Zota I.M., Abdulan I., Rusu C., Popescu R., Antoci L.M. (2022). Genetic Polymorphisms in a Familial Hypercholesterolemia Population from North-Eastern Europe. J. Pers. Med..

[17] Khera A.V., Won H.-H., Peloso G.M., Lawson K.S., Bartz T.M., Deng X., van Leeuwen E.M., Natarajan P., Emdin C.A., Bick A.G. (2016). Diagnostic Yield and Clinical Utility of Sequencing Familial Hypercholesterolemia Genes in Patients With Severe Hypercholesterolemia. J. Am. Coll. Cardiol..

[18] Bhattacharyya J., Mihara K., Bhattacharjee D., Mukherjee M. (2017). Telomere length as a potential biomarker of coronary artery disease. Indian J. Med. Res..

[19] Voight B.F., Peloso G.M., Orho-Melander M., Frikke-Schmidt R., Barbalic M., Jensen M.K., Hindy G., Hólm H., Ding E.L., Johnson T. (2012). Plasma HDL cholesterol and risk of myocardial infarction: A mendelian randomisation study. Lancet.

[20] Nelson R.H. (2013). Hyperlipidemia as a Risk Factor for Cardiovascular Disease. Prim. Care Clin. Off. Pr..

[21] Warren J.B., Dimmitt S.B., Stampfer H.G. (2016). Cholesterol trials and mortality. Br. J. Clin. Pharmacol..

[22] Zhang R., Xie J., Zhou J., Xu L., Pan Y., Qu Y., Li R., Chong M., Song L., Wen W. (2021). Supravalvular Aortic Stenosis and the Risk of Premature Death Among Patients With Homozygous Familial Hypercholesterolemia. Am. J. Cardiol..

[23] Chemello K., García-Nafría J., Gallo A., Martín C., Lambert G., Blom D. (2021). Lipoprotein metabolism in familial hypercholesterolemia. J. Lipid Res..

[24] Mabuchi H. (2017). Half a Century Tales of Familial Hypercholesterolemia (FH) in Japan. J. Atheroscler. Thromb..

[25] Do R., Project N.E.S., Stitziel N., Won H.-H., Jørgensen A.B., Duga S., Merlini P.A., Kiezun A., Farrall M., Goel A. (2014). Exome sequencing identifies rare LDLR and APOA5 alleles conferring risk for myocardial infarction. Nature.

[26] Nanchen D., Gencer B., Auer R., Räber L., Stefanini G.G., Klingenberg R., Schmied C.M., Cornuz J., Muller O., Vogt P. (2015). Prevalence and management of familial hypercholesterolaemia in patients with acute coronary syndromes. Eur. Hear. J..

[27] Mainieri F., Tagi V.M., Chiarelli F. (2022). Recent Advances on Familial Hypercholesterolemia in Children and Adolescents. Biomedicines.

[28] Raal F.J., Pilcher G.J., Panz V.R., van Deventer H.E., Brice B.C., Blom D.J., Marais A.D. (2011). Reduction in Mortality in Subjects With Homozygous Familial Hypercholesterolemia Associated With Advances in Lipid-Lowering Therapy. Circulation.

[29] Last A.R., Ference J.D., Menzel E.R. (2017). Hyperlipidemia: Drugs for Cardiovascular Risk Reduction in Adults. Am. Fam. Physician.

[30] Thompson G.R. (2021). FH through the retrospectoscope. J. Lipid Res..

[31] Mytilinaiou M., Kyrou I., Khan M., Grammatopoulos D.K., Randeva H.S. (2018). Familial Hypercholesterolemia: New Horizons for Diagnosis and Effective Management. Front. Pharmacol..

[32] Masana L., Zamora A., Plana N., Comas-Cufí M., Garcia-Gil M., Martí-Lluch R., Ponjoan A., Alves-Cabratosa L., Elosua R., Marrugat J. (2019). Incidence of Cardiovascular Disease in Patients with Familial Hypercholesterolemia Phenotype: Analysis of 5 Years Follow-Up of Real-World Data from More than 1.5 Million Patients. J. Clin. Med..

[33] Kayikcioglu M. (2021). LDL Apheresis and Lp (a) Apheresis: A Clinician’s Perspective. Curr. Atheroscler. Rep..

[34] Sharifi M., Rakhit R.D., E Humphries S., Nair D. (2016). Cardiovascular risk stratification in familial hypercholesterolaemia. Heart.

[35] Soran H., Adam S., Mohammad J.B., Ho J.H., Schofield J., Kwok S., Siahmansur T., Liu Y., Syed A., Dhage S.S. (2018). Hypercholesterolaemia – practical information for non-specialists. Arch. Med. Sci..

[36] Hartgers M.L., Besseling J., Stroes E.S., Wittekoek J., Rutten J.H., de Graaf J., Visseren F., Imholz B.P., van Lennep J.E.R., Huijgen R. (2018). Achieved LDL cholesterol levels in patients with heterozygous familial hypercholesterolemia: A model that explores the efficacy of conventional and novel lipid-lowering therapy. J. Clin. Lipidol..

[37] Arroyo-Olivares R., Alonso R., Quintana-Navarro G., Fuentes-Jiménez F., Mata N., Muñiz-Grijalvo O., Díaz-Díaz J.L., Zambón D., Arrieta F., García-Cruces J. (2019). Adults with familial hypercholesterolaemia have healthier dietary and lifestyle habits compared with their non-affected relatives: The SAFEHEART study. Public Health Nutr..

[38] Smilde T.J., Trip M.D., Wollersheim H., Van Wissen S., Kastelein J.J.P., Stalenhoef A.F.H. (2000). Rationale, Design and Baseline Characteristics of a Clinical Trial Comparing the Effects of Robust vs Conventional Cholesterol Lowering and Intima Media Thickness in Patients with Familial Hypercholesterolaemia. Clin. Drug Investig..

[39] Toth P.P., Ballantyne C.M., Davidson M.H., Tomassini J.E., Ramey D.R., Neff D., Tershakovec A.M., Hu X.H., Tunceli K. (2012). Changes in prescription patterns before and after reporting of the Ezetimibe and Simvastatin in Hypercholesterolemia Enhances Atherosclerosis Regression trial (ENHANCE) results and expected effects on low-density lipoprotein-cholesterol reduction. J. Clin. Lipidol..

[40] Zodda D., Giammona R., Schifilliti S. (2018). Treatment Strategy for Dyslipidemia in Cardiovascular Disease Prevention: Focus on Old and New Drugs. Pharmacy.

[41] Mlinaric M., Bratanic N., Dragos V., Skarlovnik A., Cevc M., Battelino T., Groselj U. (2020). Case Report: Liver Transplantation in Homozygous Familial Hypercholesterolemia (HoFH)—Long-Term Follow-Up of a Patient and Literature Review. Front. Pediatr..

[42] Lambert C.T., Sandesara P., Isiadinso I., Góngora M.C., Eapen D., Bhatia N., Baer J.T., Sperling L. (2014). Current Treatment of Familial Hypercholesterolaemia. Eur. Cardiol. Rev..

[43] Pang J., Chan D.C., Watts G.F. (2020). The Knowns and Unknowns of Contemporary Statin Therapy for Familial Hypercholesterolemia. Curr. Atheroscler. Rep..

[44] Krähenbühl S., Pavik-Mezzour I., Von Eckardstein A. (2016). Unmet Needs in LDL-C Lowering: When Statins Won’t Do!. Drugs.

[45] Kavey R.-E.W., Manlhiot C., Runeckles K., Collins T., Gidding S.S., Demczko M., Clauss S., Harahsheh A.S., Mietus-Syder M., Khoury M. (2020). Effectiveness and Safety of Statin Therapy in Children: A Real-World Clinical Practice Experience. CJC Open.

[46] Mach F., Ray K.K., Wiklund O., Corsini A., Catapano A.L., Bruckert E., De Backer G., A Hegele R., Hovingh G.K., A Jacobson T. (2018). Adverse effects of statin therapy: Perception vs. the evidence – focus on glucose homeostasis, cognitive, renal and hepatic function, haemorrhagic stroke and cataract. Eur. Hear. J..

[47] Cohen H., Stefanutti C., The Mighty Medic Satellite Research Group for Pediatric Dyslipidemia (2021). Current Approach to the Diagnosis and Treatment of Heterozygote and Homozygous FH Children and Adolescents. Curr. Atheroscler. Rep..

[48] Harada-Shiba M., Kastelein J.J., Hovingh G.K., Ray K.K., Ohtake A., Arisaka O., Ohta T., Okada T., Suganami H., Wiegman A. (2018). Efficacy and Safety of Pitavastatin in Children and Adolescents with Familial Hypercholesterolemia in Japan and Europe. J. Atheroscler. Thromb..

[49] Gallego-Colon E., Daum A., Yosefy C. (2020). Statins and PCSK9 inhibitors: A new lipid-lowering therapy. Eur. J. Pharmacol..

[50] Lagace T.A. (2014). PCSK9 and LDLR degradation. Curr. Opin. Lipidol..

[51] Guo Q., Feng X., Zhou Y. (2020). PCSK9 Variants in Familial Hypercholesterolemia: A Comprehensive Synopsis. Front. Genet..

[52] Matías-Pérez D., Pérez-Santiago A., Medina M.S., Osorno J.A., García-Montalvo I. (2021). *PCSK9* gene participates in the development of primary dyslipidemias. Balk. J. Med. Genet..

[53] Shapiro M.D., Tavori H., Fazio S. (2018). PCSK9. Circ. Res..

[54] Gareri C., Polimeni A., Giordano S., Tammè L., Curcio A., Indolfi C. (2022). Antisense Oligonucleotides and Small Interfering RNA for the Treatment of Dyslipidemias. J. Clin. Med..

[55] Merćep I., Friščić N., Strikić D., Reiner Ž. (2022). Advantages and Disadvantages of Inclisiran: A Small Interfering Ribonucleic Acid Molecule Targeting PCSK9—A Narrative Review. Cardiovasc. Ther..

[56] Ray K.K., Stoekenbroek R.M., Kallend D., Nishikido T., Leiter L.A., Landmesser U., Wright R.S., Wijngaard P.L.J., Kastelein J.J.P. (2019). Effect of 1 or 2 Doses of Inclisiran on Low-Density Lipoprotein Cholesterol Levels. JAMA Cardiol..

[57] Bin Saleh F.S., Alharbi W.S., Alanazi G.B., Aldughaither A. (2022). Prevalence and Regulation of Dyslipidemia Among Adults With Type 2 Diabetes From Three Primary Health Care Centers in Riyadh. Cureus.

[58] Raal F.J., Kallend D., Ray K.K., Turner T., Koenig W., Wright R.S., Wijngaard P.L., Curcio D., Jaros M.J., Leiter L.A. (2020). Inclisiran for the Treatment of Heterozygous Familial Hypercholesterolemia. New Engl. J. Med..

[59] Ray K.K., Wright R.S., Kallend D., Koenig W., Leiter L.A., Raal F.J., Bisch J.A., Richardson T., Jaros M., Wijngaard P.L. (2020). Two Phase 3 Trials of Inclisiran in Patients with Elevated LDL Cholesterol. New Engl. J. Med..

[60] Chiang J.Y.L. (2013). Bile Acid Metabolism and Signaling. Compr. Physiol..

[61] Davidson M. (2013). The Efficacy of Colesevelam HCl in the Treatment of Heterozygous Familial Hypercholesterolemia in Pediatric and Adult Patients. Clin. Ther..

[62] Yamashita S., Arai H., Bujo H., Masuda D., Ohama T., Ishibashi T., Yanagi K., Doi Y., Nakagawa S., Yamashiro K. (2021). Probucol Trial for Secondary Prevention of Atherosclerotic Events in Patients with Coronary Heart Disease (PROSPECTIVE). J. Atheroscler. Thromb..

[63] Yamashita S., Matsuzawa Y. (2009). Where are we with probucol: A new life for an old drug?. Atherosclerosis.

[64] Vaezi Z., Amini A. (2022). Familial Hypercholesterolemia. StatPearls.

[65] Szczepańska E., Białek-Dratwa A., Janota B., Kowalski O. (2022). Dietary Therapy in Prevention of Cardiovascular Disease (CVD)—Tradition or Modernity? A Review of the Latest Approaches to Nutrition in CVD. Nutrients.

